# Investigating the Metabolism of Estrogens in *Ligilactobacillus salivarius* Strains Isolated from Human Milk and Vaginal Microbiota

**DOI:** 10.3390/nu16060861

**Published:** 2024-03-16

**Authors:** Alberto Aragón, Rubén Jurado, Josué Jara, Juan Miguel Rodríguez, Belén Orgaz

**Affiliations:** 1 Department of Galenic Pharmacy and Food Science, School of Veterinary Sciences, University Complutense of Madrid (UCM), 28040 Madrid, Spain; alberara@ucm.es (A.A.); rubenjur@ucm.es (R.J.);; 2 Department of Nutrition and Food Science, School of Veterinary Sciences, University Complutense of Madrid (UCM), 28040 Madrid, Spain

**Keywords:** estrone, 17β-estradiol, *Ligilactobacillus salivarius*, bacteria-degrading estrogens, probiotics

## Abstract

The interplay between enterohepatic circulation and the gut microbiota is the main driver determining systemic levels of estrogens and their metabolites. Nevertheless, the role of potentially probiotic microorganisms in estrogen metabolism has not been investigated so far. In this work, we have explored the ability of six *Ligilactobacillus salivarius* strains isolated from human milk and vaginal samples to degrade and/or conjugate parental estrogens in vitro and under aerobic conditions. The quantification of estrogens and their derivatives was carried out in cell-free supernatants by LC-QQQ-MS. All the tested *L. salivarius* strains achieved an average degradation rate of estrone and estriol of 98% and 55%, respectively, whereas 17β-estradiol was preferentially conjugated (up to 40%). The presence of seven out of ten genes encoding enzymes relevant for estrogen metabolism was further confirmed by PCR, highlighting their genetic potential for degrading, conjugating and/or deconjugating estrogens. The tested *L. salivarius* strains may be considered potential probiotics affecting the fate of endogenous estrogens. Clinical trials targeting populations with estrogen-dependent conditions will be required to elucidate the true potential of these strains for the restoration and maintenance of a healthy host estrobolome.

## 1. Introduction

The role of estrogens in the development and function of the female reproductive tract is well known [1,2]. These hormones play a pivotal role in the creation of a healthy microenvironment in the lower reproductive tract through a wide variety of mechanisms, from the regulation of epithelial thickness, glycogen levels and mucus secretion to the promotion of lactobacilli populations and lactic acid production [3,4,5].

In addition, estrogens display key pleiotropic effects on non-reproductive organs (e.g., the gut, the brain, bones, mammary glands) and tissues (e.g., adipose tissue) [1,6,7,8,9]. As a consequence, they are essential for critical physiological processes, including glucose homeostasis, bone and cardiovascular health, and immune and neural functions [10,11,12,13,14]. However, estrogens also participate, directly or indirectly, in most human pathologies, including infectious, autoimmune, metabolic, oncologic and degenerative diseases [15,16,17]. Menopause and post-menopause are life stages particularly predisposed to estrogen-related disorders and diseases as a result of the depletion of circulating estrogens [3,18,19,20,21,22].

In recent decades, the pivotal role of the gut microbiota in host health has become a growing area of research [23]. The impact of the gut microbiome extends beyond the gut since some microbes are able to translocate under physiological conditions but, most interestingly, because many microbial metabolites, components and enzymes are absorbed and systemically distributed throughout the body [24,25]. In turn, several factors associated with the host and its environment may exert a strong influence on the composition of the gut microbiome [26,27,28,29,30]. In this regard, there is a bidirectional relationship between the gut microbiome and estrogens since the composition of the gut microbiota is modulated by these hormones, while the gut microbiome significantly influences systemic estrogen levels [31,32,33]. A wide range of compounds, including estrogens, are conjugated in the liver and gut as a part of their metabolism via UDP-glucuronosyltransferase (UGT) and sulfotransferase (SULT) enzymes [34,35]. These enzymes are in charge of transferring glucuronic residues and sulfate groups to the parental estrogens, respectively. Their conjugated forms are metabolically inactive and more soluble, and they can be excreted in bile, urine and feces. However, a significant proportion of these inactive forms are reabsorbed through enterohepatic circulation [36]. This process is mediated by microbial enzymes, including β-glucosidases [37] and, especially, β-glucuronidases [31]. The latter enzymes deconjugate estrogens, allowing these hormones to bind to estrogen receptors (ERs) and elicit their physiological effects [32,38]. Conjugation–deconjugation equilibrium is essential, as conjugated estrogens are not appreciable ligands for ERs and, thus, they do not promote ER-mediated activity. This fact is very relevant for health since both an excess and a deficiency of active estrogens play an important role in a wide variety of gynecologic-related (endometriosis, polycystic ovary syndrome, infertility, etc.) and non-gynecologic-related diseases, such as those cited above [12,32,39,40,41,42].

In this context, the study of the estrobolome, which is defined as the repertoire of genes of the gut microbiota capable of metabolizing estrogens [38], has recently acquired great interest [32,43,44,45,46]. Knowledge about estrogen metabolism by autochthonous members of the human microbiota is very scarce at present. However, improvements in this field would allow for the targeted selection of probiotic strains for applications in some of the pathologies mentioned above. The objective of this work is to investigate the potential of six *Ligilactobacillus salivarius* strains isolated from either human milk or vaginal samples to participate in estrogen metabolism. The knowledge gained from this work could pave the way for using lactobacillus strains to modulate estrogen levels, either independently or in combination with conventional anti-estrogenic therapies, to mitigate or ameliorate the symptoms associated with estrogen-related gynecological conditions such as endometriosis.

## 2. Materials and Methods

### 2.1. Bacterial Strains and Culture Conditions

Six *L. salivarius* strains were used in this study. They were isolated and identified by sequencing the 16S rRNA gene in previous work by the group from either human milk or vaginal samples (Table 1) [47,48]. The strains were grown aerobically in De Man, Rogosa and Sharpe (MRS, Oxoid, Basingstoke, UK) medium at 37 °C for 24 h under static conditions from frozen stock cultures kept in glycerol (1.5% *v*/*v*). Pre-cultures and cultures were prepared in the same medium for 24 h, and the bacterial concentration of the cellular suspensions was determined by plating on MRS agar plates. Plates were incubated at 37 °C for 48 h for enumeration, and the results were expressed as colony-forming units (cfu) per milliliter (cfu/mL).

### 2.2. Bacterial Growth in the Presence of Estrogens

Estrone (E1), 17β-estradiol (E2) and estriol (E3) were obtained from Agilent Technologies Inc. (Santa Clara, CA, USA). Stock solutions of each estrogen were prepared to reach a stock concentration of 0.1 M in 100% ethanol (Sigma Aldrich, Madrid, Spain, HPLC purity). Subsequently, 10 μL of these stock solutions were added to MRS broth tubes (990 μL), yielding a final concentration of primary estrogens in the culture medium of 10^−4^ M. Such concentrations were equivalent to 26, 30 and 28 ppm of E1, E2 and E3, respectively, depending on the molecular weight of each estrogen. To evaluate the ability of the selected strains to metabolize estrogens, estrogen-containing media were inoculated with the amount of an overnight culture required to obtain an initial concentration of 10^5^ cfu/mL. The tubes were incubated aerobically at 37 °C for 48 h in static conditions. After incubation, cultures were centrifuged (7500× *g*, 4 °C, 20 min). Supernatants were recovered, filtered through an acetate filter (0.22 μm) and kept at −80 °C to avoid oxidative reactions until use. The absence of an antimicrobial effect of ethanol on the growth of the strains of *L. salivarius* was confirmed previously by growing the *L. salivarius* strains in MRS containing the same concentrations of ethanol but devoid of estrogens. In parallel, assays were performed using either non-bacterial inoculated culture medium supplemented with the three estrogens or bacterial cultures in MRS broth devoid of estrogens in order to serve as controls to discard estrogen degradation due to the ingredients of the culture medium and/or the production of bacterial metabolites that could interfere with the detection of estrogens and their metabolites.

### 2.3. Extraction of Estrogens

The extraction of free (F) and total (T; free and conjugated) estrogens was carried out from the cell-free supernatants according to the protocol described by Xu et al. [49] with some modifications. For F-estrogen extraction, 0.5 mL of the cell-free supernatants were mixed with 1 mL of dichloromethane (Sigma Aldrich, Madrid, Spain, HPLC quality) and subjected to a slow reverse extraction process (flipping the tubes 360°) for 30 min at 50 rpm. The slow extraction preserves the highly oxidizable lipid fractions, avoiding the generation of air bubbles and damage to these compounds. This extraction process was repeated three times. Then, the total aqueous fraction was discarded, and the solvent was evaporated using a stream of nitrogen to recover the organic fraction. The same procedure was used for the extraction of T-estrogens, although, in this case, a previous enzymatic hydrolysis step was introduced to release the conjugated estrogens (C-estrogens). For this purpose, 0.5 mL of enzymatic hydrolysis buffer containing 2 mg of L-ascorbic acid, 5 μL of β-glucuronidase/sulfatase (Sigma Aldrich, Madrid, Spain) and 0.5 mL of 0.15 M acetate buffer (pH 4.1) was added to 0.5 mL of the cell-free supernatants. The reaction mixture was incubated at 37 °C for 20 h. After this step, the extraction procedure was continued as described above. The extraction of F- and T-estrogens was also carried out from the control samples.

For each estrogen (E1, E2 and E3), the concentration of conjugated derivates was calculated as the difference between the total estrogen value and the free estrogen value. The concentration of metabolized or consumed estrogen was calculated as the difference between the initial concentration of each estrogen in the broth media and the total concentration value remaining in the media after 48 h of incubation. Metabolization/consumption rates and conjugation rates were calculated for each estrogen.

### 2.4. Determination of Estrogens and Derivatives by Triple-Quadrupole Mass Spectrometry Coupled with High-Performance Liquid Chromatography (LC-QQQ-MS)

Commercial standards of primary estrogens and some of their estrogenic derivatives that may exhibit biological activity (estrone, estriol, 17β-estradiol, 2-hydroxy-estrone-3-methyl-ether, 2-methoxy-estradiol, 4-methoxy-17β-estradiol, 4-methoxy-estrone, 16α-hydroxy-estrone, 2-hydroxy-estrone, 4-hydroxy-estrone, 17-epi-estriol, 2-methoxy-estrone) were purchased from Agilent Technologies Inc. (Santa Clara, CA, USA) and reconstituted in 100 μL of methanol. The quantification of parental estrogens and derivates was carried out using LC-QQQ-MS (LCMS-8030, Shimadzu, Kyoto, Japan) equipment at the facilities of the Spectrophotometry Center of Complutense University of Madrid (Madrid, Spain), according to the protocol described by Xu et al. [47] with some modifications. Briefly, calibration samples were made up at different concentrations of each compound in the range of 1–100 mg/L to establish the limit of quantification (LOQ) and the limit of detection (LOD) for each analyte (Table 2). The volume of reconstituted sample injected was 10 μL. In this study, we used a 2.1 × 50 mm, 2.7 μm Poroshell 120 PhenylHexyl column (Agilent Technologies Inc, Santa Clara, CA, USA). Phase A was composed of H_2_O and 0.1% formic acid, and phase B was composed of methanol and 0.1% formic acid. The following parameters (percentage of each mobile phase and initial time) were used for the HPLC run: 5% phase B, 1 min; 40% phase B, 11 min; 64% phase B, 12 min; 95% phase B, 13 min; 95% phase B, 14 min; and 5% phase B, final. The flow rate used was 0.5 mL/minute, and the total run time was 17 min. For selective quantification, the dynamic MRM mode (MRM transitions) was used, which improves the sensitivity to resolve compounds that, as happens with estrogen derivatives, have similar retention times (Table 2).

### 2.5. PCR Detection of Estrogen Metabolism-Related Enzymes

The presence of genes encoding estrogen metabolism-related enzymes (α-glucosidase, β-glucosidase, α-glucuronidase, β-glucuronidase, glucosyltransferase, sugar transferase, bifunctional acetaldehyde-CoA/alcohol dehydrogenase, 3-hydroxyacyl-CoA dehydrogenase, acyl-CoA thioesterase, and short-chain dehydrogenase) was screened using PCR using the primers described in Table 3. The primer pairs targeting the first 6 genes were designed on the basis of the *L. salivarius* genomes available in databanks using the primer design software Primer3 (https://primer3.ut.ee/, accessed on 24 November 2023) and were analyzed and optimized using the oligonucleotide sequence calculator OligoAnalyzer™ Tool (https://www.idtdna.com/calc/analyzer, accessed on 24 November 2023). In silico PCR amplification was performed to confirm that the designed primer pairs would anneal to their respective gene targets in the *L. salivarius* published genomes (http://insilico.ehu.es/PCR/, accessed on 24 November 2023). Primer pairs designed in previous works were employed to amplify the genes encoding bifunctional acetaldehyde-CoA/alcohol dehydrogenase, 3-hydroxyacyl-CoA dehydrogenase, acyl-CoA thioesterase and short-chain dehydrogenase [50,51]. PCR amplifications were performed in an iCycler^®^ thermocycler (Bio-Rad, Hercules, CA, USA). Each PCR reaction mixture consisted of 12.5 µL of BioMix™ Red 2× (Ecogen, Barcelona, Spain), 6.5 µL of nuclease-free water (Sigma Aldrich, Madrid, Spain), 0.5 µL of each primer (10 mM) (Table 3) and 5 µL of the isolated genomic DNA from each *L. salivarius* strain according to the protocol described by Baele et al. [52]. The PCR amplification conditions were as follows: one cycle of 95 °C for 5 min, 30 cycles of 95 °C for 30 s, 55 °C for 30 s and 72 °C for 1 min, and a final extension of 72 °C for 5 min. Finally, PCR products were subjected to 1.2% (*w*/*v*) agarose gel electrophoresis. Gels were run using a PowerPac Basic (Bio-Rad) and a Wide mini-subcell GT (Bio-Rad) for 30 min at 90 V. Gel Red^®^ Nucleic Acid Stain (Biotium, Fremont, CA, USA) (1:100) was used to visualize the DNA bands, and the gels were observed in a Bio-Rad Universal Hood II transilluminator (Bio-Rad). Gel images were acquired using a Gel Doc 1000 Documentation System software, version 4.6.9 (Bio-Rad).

### 2.6. Statistical Analysis

Three independent experiments were performed for each assay to obtain mean values. Data were analyzed with Statgraphics Centurion software, version 18.1.06 (Statistical Graphics Corporation, Rockville, MD, USA). A one-way analysis of variance (ANOVA) was carried out to determine whether samples were different or not at a 95% confidence level (*p* < 0.05).

## 3. Results

### 3.1. Ligilactobacillus salivarius Strains Can Degrade and Conjugate Parental Estrogens

In this study, we evaluated the ability of six *L. salivarius* strains to degrade and/or conjugate the three major naturally occurring estrogens, estrone (E1), 17β-estradiol (E2) and estriol (E3). Initially, we tested if the addition of 10 μL of methanol to MRS broth tubes (990 μL) exerted any influence on the growth of the strains when incubated at 37 °C for 48 h since the stock solutions of the three estrogens were made in 100% methanol. The addition of this quantity of methanol did not modify the concentration of any of the three strains when compared with cultures in the same medium without methanol supplementation. Moreover, we did not detect any of the estrogens in the bacterial cultures growing in MRS. Similarly, in the estrogen-containing MRS broth without bacteria incubated at 37 °C for 48 h, parental estrogen degradation was not detected.

**Figure 1:**
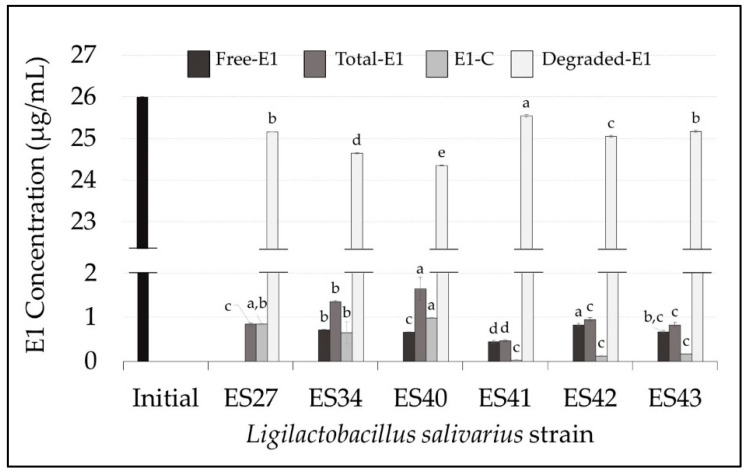
Concentration (µg/mL) of free, total, conjugated and degraded estrone (E1) in the cell-free supernatants of the *L. salivarius* strains included in this study. E1 initial concentration: 26 µg/mL (black bar). Values are expressed as the mean ± SD (n = 3). Conjugated E1 (E1-C): total E1–free E1; degraded E1: initial E1–total E1. Different superscript letters in the same bar color for the different *L. salivarius* strains mean statistically significant differences (*p* < 0.05).

Subsequently, the strains were incubated in estrogen-containing MRS broth. All of them were able to grow in the presence of the three estrogens, reaching an average population density of (7.7 ± 0.6) log_10_ cfu/mL. The levels of free, total (free + conjugated) and degraded E1 in the cell-free supernatants are shown in Figure 1.

**Figure 2:**
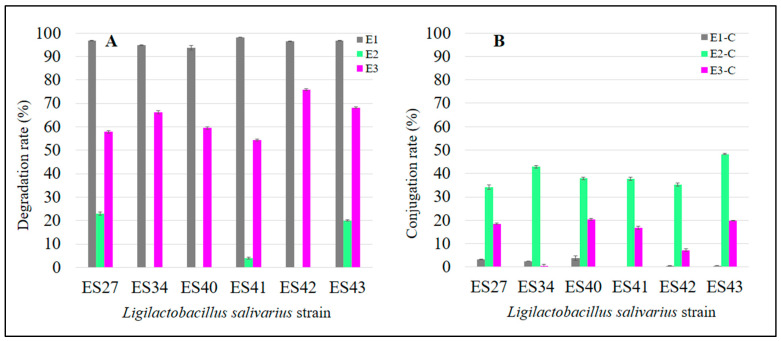
Degradation (**A**) and conjugation (**B**) rates of parental estrogens (E1, E2 and E3) after the incubation of the *L. salivarius* cultures for 48 h at 37 °C.

After 48 h of incubation at 37 °C, all the *L. salivarius* strains tested were able to drastically reduce the initial level of E1. The residual mean values of total E1 in the broth medium ranged from 0.46 ± 0.03 µg/mL (*L. salivarius* ES41) to 1.65 ± 0.26 µg/mL (*L. salivarius* ES40). This means an E1 degradation rate ranging from 97% to 99% (Figure 2A).

The E1-degrading ability of *L. salivarius* ES27 (vaginal origin), ES41 and ES43 (human milk origin) was significantly higher (*p* < 0.05) than that exhibited by the rest of the strains (Figure 1). Moreover, *L. salivarius* ES27, ES34 and ES40 were able to conjugate (i.e., transfer a glycosyl residue to the parental estrogen) approximately 3% of the parental E1 initially added (Figure 2B). The biotransformation of E1 to other primary estrogens was not detected in this study.

**Figure 3:**
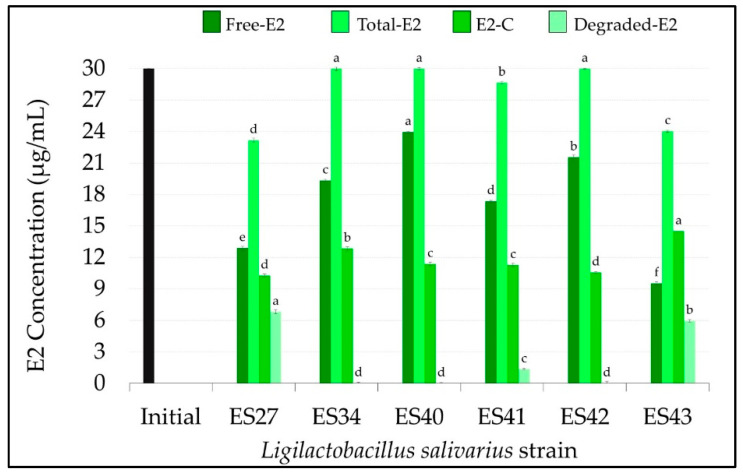
Concentration (µg/mL) of free, total, conjugated and degraded 17β-estradiol (E2) in the cell-free supernatants of the *L. salivarius* strains included in this study. E1 initial concentration: 30 µg/mL (black bar). Values are expressed as the mean ± SD (n = 3). Conjugated (E2-C): total E2–free E2; degraded E2: initial E2–total E2. Different superscript letters in the same bar color for the different *L. salivarius* strains mean statistically significant differences (*p* < 0.05).

After 48 h of incubation at 37 °C, only three *L. salivarius* strains were able to degrade E2, these being strains ES27 and ES43, which exhibit the highest degradation rates (*p* < 0.05) (Figure 2A and Figure 3). These strains consumed 6.82 ± 0.18 µg/mL and 5.96 ± 0.14 µg/mL of E2, respectively, which means a degradation of ~20% of the free E2 initially added (Figure 2A and Figure 3).

All the strains were able to conjugate this estrogen, although the conjugation ability was strain-dependent. While most of the non-degraded E2 remained in its free form in the cell-free supernatants of strains ES40 and ES42 (23.96 ± 0.08 and 21.55 ± 0.21 µg/mL, respectively), *L. salivarius* ES27, ES41 and ES43 were able to conjugate ~ 40% of the initial free E2 concentration (Figure 2B and Figure 3). Interestingly, *L. salivarius* ES27, ES43 and, to a lesser extent, ES41 were the only strains that exhibited the ability to both degrade and conjugate this estrogen (Figure 3).

**Figure 4:**
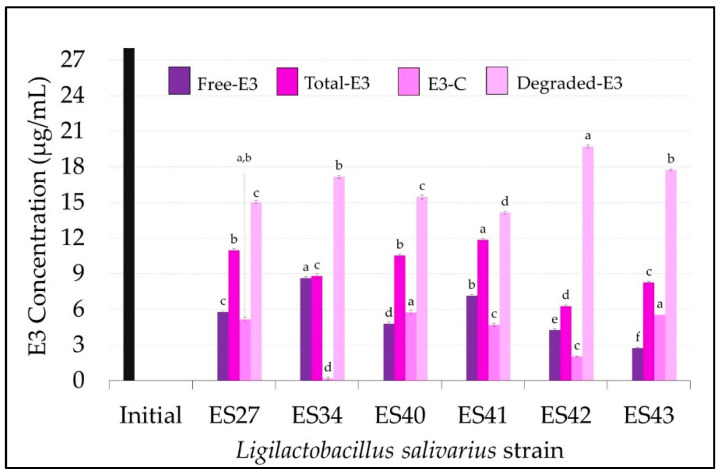
Concentration (µg/mL) of free, total, conjugated and degraded estriol (E3) in the cell-free supernatants of the *L. salivarius* strains included in this study. E3 initial concentration: 27 µg/mL (black bar). Values are expressed as the mean ± SD (n = 3). Conjugated (E3-C): total E3–free E3; degraded E3: initial E3–total E3. Different superscript letters in the same bar color for the different *L. salivarius* strains mean statistically significant differences (*p* < 0.05).

Finally, the levels of free and total E3 in the cell-free supernatants of the *L. salivarius* strains are shown in Figure 4. The results achieved by the different strains with E3 showed a higher degree of variability when compared with the previous estrogens (E1 and E2). *L. salivarius* ES42 and ES43 showed higher estriol degradation rates (*p* < 0.05) than the rest of the strains tested in this study, consuming 19.72 ± 0.12 µg/mL and 17.74 ± 0.09 µg/mL of E3, respectively, which means degradation rates of 76% and 68%, respectively (Figure 2A and Figure 4). The E3 conjugation rates differed notably among the *L. salivarius* strains. Whereas most of the non-consumed E3 remained in its free form in the supernatant of the strain ES34 (8.64 ± 0.13 µg/mL) (Figure 4), strains ES27, ES40 and ES43 seemed to have more effective conjugation mechanisms for this specific estrogen since ~20% of the remaining E3 in their supernatants was conjugated (Figure 2B). The biotransformation of E3 to other estrogen derivates was undetected in this study.

### 3.2. Ligilactobacillus salivarius Strains Isolated from Human Milk and Vaginal Samples Exhibit Genetic Capacity for Estrogen Degradation

To assess whether our strains harbor the genetic potential for estrogen degradation and/or participation in conjugation/deconjugation mechanisms, we first searched in the literature for relevant enzymes involved in these processes and selected ten of them, belonging to three categories: enzymes related to estrogen conjugation/deconjugation mechanisms, enzymes involved in the degradation of the steroid ring and enzymes degrading the side chain of estrogens (Table 3). The presence of genes encoding seven of them in the tested strains was confirmed using PCR (Table 4). None of the strains harbored genes encoding β-glucuronidase (*gusA*, *uidA*), although all of them presented genes related to β-glucosidase activity (*bglH*). Additionally, all the strains exhibited genes encoding enzymes belonging to the glycosyltransferase family (GT), implicated in the transference of glycosylated residues to various substrates, including estrogens, to make them more soluble. Regarding enzymes degrading the steroid ring, all our strains harbored genes encoding various oxidoreductases, acetaldehyde-CoA/alcohol dehydrogenases and 3-hydroxyacyl-CoA dehydrogenases (*adhE*, *FadB/echA*), which are involved in the degradation pathways of aromatic compounds through successive oxidations of their lateral residues. Furthermore, all the strains carried genes encoding two enzymes (acyl-CoA thioesterase and short-chain dehydrogenase) participating in the degradation of the side chain of steroids, driving successive oxidations of the C-C side chain similar to those occurring in the β-oxidation of fatty acids for energy production.

## 4. Discussion

The interplay between enterohepatic circulation and the gut microbiota is the main driver determining systemic levels of estrogens and their metabolites. Although a few studies have addressed the potential role of gut microbes in estrogen conjugation–deconjugation mechanisms in pathological conditions, such as the pathogenesis of breast cancer [53], the ability of probiotic bacteria to degrade and/or conjugate estrogens has not been investigated so far.

In this work, we have explored the potential role of six *L. salivarius* strains isolated from samples of human milk and vaginal samples provided by healthy women to degrade and/or conjugate parental estrogens in vitro. Overall, all the tested *L. salivarius* strains could be considered good candidates for degrading estrone and estriol. Although slight strain-associated differences were observed, all of them reached a degradation (metabolization) rate of around 90% and 50% of the initial concentrations of estrone and estriol, respectively. It must be highlighted that only three out of the six *L. salivarius* strains were able to degrade 17β-estradiol, these being strains ES27 and ES43, which exhibited the highest degradation rates.

The carbon backbone of steroids consists of four fused rings (named A to D) carrying different side substitutions (alcohol, ketone groups and carbon chains of up to ten carbon atoms). The oxidation of the steroid core and the degradation of side chains according to the fatty acid oxidation pathway are key reactions for their microbial degradation [54,55]. Although all our *L. salivarius* strains harbor seven genes encoding enzymes participating in estrogen metabolism, their capacity to degrade E2 differed significantly, suggesting that they may exhibit different regulatory pathways, as previously reported [56]. Moreover, the E2-degrading ability of our strains was low (~20%) when compared with their capacity to degrade E1 and E3. Several studies have suggested that the conversion of E2 into E1 is the initial step for effective E2 degradation [57,58], a process in which the enzyme 17β-hydroxysteroid dehydrogenase (17β-HSD) (EC 1.1.1.51) catalyzes the dehydrogenation at the C-17 position of the D ring of E2. In this work, genes codifying for two dehydrogenases acting on the CH-OH group of donors in a way similar to 17β-HSD (*adhE* and *FadB/echA*) were detected in the tested strains. Similarly, Xiong et al. [59] demonstrated that the presence of E2 was able to induce the expression of four dehydrogenases similar to 17β-HSD in *Stenotrophomonas maltophilia* SJTH1, oxidizing up to 90% of E2 into E1. Similarly, Wang et al. [60] demonstrated that *Pseudomonas putida* SJTE-1 possesses the enzyme 3-oxoacyl-(acyl-carrier protein) reductase, an enzyme capable of functioning as 17β-HSD. This enzyme could convert 17β-estradiol into estrone, showing a transformation efficiency higher than 96%. All these enzymes, including those present in our strains (EC 1.1.1.1 and EC 1.1.1.35), belong to the oxidoreductase family. Although substrate specificity is variable among them, these works suggest that, under substrate induction, different enzymes of this family can catalyze the conversion of E2 into E1, although in our case, to a lesser extent. Other degradation pathways of E2 that do not involve the initial conversion into E1 have been proposed for *Lactobacillus casei* LC-1, although the exact mechanisms have not been elucidated yet [55]. It should be highlighted that estrogen-degrading mechanisms have been widely studied in microorganisms isolated from soil, sewage and water, where the concentration of environmental estrogens may be relatively high [61,62]. Contrary to our results, most of the bacteria-degrading estrogens isolated from the above-mentioned sources are especially active towards 17β-estradiol, the most abundant estrogen in environmental samples [63]. This fact suggests that environmental microorganisms have developed more effective pathways for degrading or transforming this specific estrogen. In contrast, our human-origin strains seem to favor conjugation over the degradation of 17β-estradiol.

Estradiol is the main intracellular estrogen in women, and its estrogenic potential is approximately 50 and 6 times more potent than those of estrone and estriol, respectively [64]. Under physiological conditions, the systemic estrone and estradiol levels in women at a reproductive age range between 5 and 31 μg and between 3 and 19 μg per day, respectively. Some pathologic conditions, such as premature ovarian failure (POF), and aging may lower these levels, leading to metabolic imbalances and tissue degeneration [14], and the values may be particularly low in menopause and post-menopause women [22,65,66]. On the other hand, endometriosis is an estrogen-dependent disease characterized by the presence of endometrial tissue outside of the uterine cavity. Estrogen levels are increased in this condition, especially those of 17β-estradiol, as endometriotic tissue is deficient in the enzyme 17β-HSD [67]. The fact that the *L. salivarius* strains tested in this study are especially prone to conjugate 17β-estradiol and, also, carry the genes encoding two dehydrogenases that act in a way similar to that of 17β-HSD suggests that they may have potential for preventing the above-mentioned estrogen-related gynecological pathologies.

A wide range of compounds, including hormones, environmental pollutants, neurotransmitters, and drugs, are conjugated in the liver or gut via UDP-glucuronosyltransferase (UGT) enzymes [34]. To the best of our knowledge, the capacity of *L. salivarius* to transfer glucuronic groups to estrogens has not been described yet. This suggests other mechanisms by which our strains may conjugate estrogens and, in particular, estradiol. In general, lactic acid bacteria have a highly conserved pathway for the synthesis of exopolysaccharides that contains a region in which glycosyltransferase (GT) genes are included. These enzymes catalyze the transference of glycosyl residues to a lipid carrier or to a growing polysaccharide chain [68]. Some genes encoding glycosyltransferases (*gtf3/csbB/epsF*) and a sugar transferase (*epsL*) were detected in our *L. salivarius* strains, but the exact mechanism by which our strains preferentially conjugate E2 should be elucidated in future studies.

In its conjugated form, estradiol is biologically inactive and unable to interact with ERs, suggesting that these *L. salivarius* strains modulate the effect of this estrogen by blocking its action until required, probably through balancing conjugation–deconjugation mechanisms. Genes related to the enzyme β-glucuronidase (*gusA*, *uidA*) could not be detected in any of the *L. salivarius* strains, although all of them harbored genes related to β-glucosidase activity. Both enzymes belong to the family of glycoside hydrolases (GH), and they cleave glucuronic acid and sugar moieties from the non-reducing end of glycosides, respectively [69]. We cannot discard that enzymes belonging to this family might hydrolyze, although to a lesser extent, other substrates. For instance, the β-glucosidase produced by *Lactobacillus casei* ATCC 393 was active against different substrates with (1,4-β) and (1,4-α) linkage configurations, including alkyl- and aryl-glucosides, cyanoglucosides and others [70].

Nowadays, it is generally accepted that bacterial glucuronidases are a part of the physiological estrobolome; however, the presence of this enzymatic activity was considered a negative trait for the selection of probiotic bacteria in the past since it was thought that an overabundance of bacteria exhibiting such activity could contribute to exacerbate estrogen-dependent pathological processes [42,71]. Nevertheless, this traditional view was somehow biased since many studies describing this activity were performed on pathobionts [67,72]. However, β-glucuronidases are ubiquitous enzymes that have been found in a variety of microorganisms, including lactic acid bacteria, with a qualified presumption of safety and/or a generally recognized as safe status [73,74]. Recently, Ervin et al. [44] have demonstrated that the administration of fecal samples rich in these enzymatic activities to mice did not correlate with an increase in tumorogenesis, suggesting that the function of these enzymes could be a way of reactivating estrogens that can be further metabolized in different ways (e.g., hydrolyzed, epimerized, oxidized, methylated) in distal sites. In parallel, Beck et al. [75] demonstrated that treatment with β-glucuronidase inhibitors did not prevent breast cancer in mice.

One of the limitations of this study is that the assays were performed under aerobic conditions. Changes in the gut bulk of glycosyl hydrolase enzymatic activities have been reported in response to changes in oxygen concentration. For instance, Nakamura et al. [76] found higher values of β-glucosidase and β-glucuronidase activities in different genera belonging to the phyla *Bacteroidota* and *Bacillota* when growing anaerobically. Moreover, our results need to be confirmed in the future, considering potential interactions between our *L. salivarius* strains and other microbiota members. Therefore, clinical trials targeting populations with estrogen-dependent conditions are required to elucidate the true potential of these strains. As stated by Plottel and Blaser [38], if such interventions prove successful, these strains could contribute to the restoration and maintenance of a healthy host estrobolome.

## 5. Conclusions

Although the results of this study were obtained using in vitro assays, they suggest that these *L. salivarius* strains may have probiotic potential in the fate of endogenous estrogens. Among them, *L. salivarius* ES27 and ES43 were able to degrade and conjugate 17β-estradiol, the most potent estrogenic compound. The knowledge gained from this work could pave the way for using lactobacillus strains to modulate estrogen levels, either independently or in combination with conventional anti-estrogenic therapies, to mitigate or ameliorate the symptoms associated with estrogen-related gynecological conditions.

## Figures and Tables

**Table 1:** Microorganisms used in this work and origin.

Microorganism	Strain Code	Origin
*Ligilactobacillus salivarius*	ES27	Vagina
*L. salivarius*	ES34	Vagina
*L. salivarius*	ES40	Human milk
*L. salivarius*	ES41	Human milk
*L. salivarius*	ES42	Human milk
*L. salivarius*	ES43	Human milk

**Table 2:** Tandem mass spectrometry-selected reaction monitoring conditions for parental estrogens and metabolites quantification. RT: retention time; CE: collision energy (V); LOD: limit of detection; LOQ: limit of quantification.

Estrogen	RT (min)	Q1	CE (V)	Q3	LOD (mg/L)	LOQ (mg/L)
Estrone	7.30	271.2	−13	253.05	0.03	0.10
			−25	133.10		
17β-Estradiol	6.70	273.0	−27	107.10	0.86	2.88
			−18	135.00		
Estriol	3.10	289.1	−25	107.00	0.03	0.09
			−14	253.05		
2-Hydroxyestrone-3-methyl ether	8.00	301.0	−18	186.95	0.01	0.02
			−19	240.20		
2-Methoxyestradiol	7.30	303.2	−24	137.00	0.01	0.04
			−19	135.20		
4-Methoxy-17β-estradiol	6.98	303.2	−25	137.05	0.06	0.18
			−20	135.20		
4-Methoxyestrone	7.60	301.0	−14	283.20	0.02	0.07
			−23	163.00		
16ἀ-Hydroxyestrone	4.40	287.0	−15	251.15	0.01	0.04
			−20	199.10		
2-Hydroxyestrone	6.00	287.0	−13	269.25	0.02	0.06
			−14	175.05		

**Table 3:** Estrogen degrading-related enzymes and PCR primer sequences.

Enzyme (EC Number)	Gene (ID)	Primer Sequence (5′-3′)	Annealing Temperature (°C)	Amplicon Size (pb)	References
α-glucosidase (3.2.1.20)	ygjK (BQ1177_RS04000)	F: GGACGATGTGGAGGAGCTAA	55.0	205	
		R: CCCAAATGCGGAACCATGAT			
β-glucosidase (3.2.1.86)	bglH (BQ1177_RS09625)	F: AATGGCCTTGGTGCAAAAGA	55.0	184	
		R: AATTTCGCCGGAACTAGCAC			
α-glucuronidase (3.2.1.139)	H0A38_RS06600	F: TACGCTCGCTTTCTTGCTTC	55.0	242	
		R: TGTTGCCACCATGAAGACAC			
β-glucuronidase (3.2.1.31)	gusA, uidA (FC33_RS02420)	F: AATTCACCGCCCCGTTAAAC	55.0	194	
		R: ATGAGTTTCCCTGAACCCGT			
Glycosyltransferase (2.4.-.-)	gtf3/csbB/epsF (BQ1177_RS07995)	F: AAGAAAATGGTGGGTTGTCTGA	55.0	100	
		R: TGACTTTCAACGTAATCATCGGA			
Sugar transferase (2.7.8.-)	epsL (BQ1177_RS00420)	F: CAAGCTCGGAGACATGAAGT	55.0	204	
		R: TGTTGCTGAACCTTCTTCTGA			
Bifunctional acetaldehyde- CoA/alcohol dehydrogenase (1.2.1.10/1.1.1.1)	adhE (BQ1177_RS09345)	F: GCATCTGACTTCACACGTCC	55.0	219	[50]
		R: TCCAAATTCCCCACCAGTCT			
3-hydroxyacyl-CoA dehydrogenase (1.1.1.35)	FadB/echA (BQ1177_RS04965)	F: AGGGGTTGCAGATCCAATGA	55.0	205	[50]
		R: GCCAGCTTCAATGCCGTAAT			
Acyl-CoA thioesterase (3.1.2.-)	paaI (BQ1177_RS04090)	F: ACTAGCCATCGTGTTTTAGCT	55.0	207	[50]
		R: GCCACTAACGTATGATTCGATAC			
Short-chain dehydrogenase(1.3.-.-)	DltE (BQ1177_RS00925)	F: GGAAGAAGTGGGACCTGTCT	55.0	172	[51]
		R: ACCTACTTGTCTTTCGGCCA			

**Table 4:** Steroid-degrading enzymes in the *Ligilactobacillus salivarius* strains selected in this work. Presence (+) or absence (−) of the enzyme-coding gene (see Table 3).

Category	Enzyme	*Ligilactobacillus salivarius* Strain					
		ES27	ES34	ES40	ES41	ES42	ES43
Conjugation/deconjugation-related enzymes	α-glucosidase	+	+	+	+	+	+
	β-glucosidase	+	+	+	+	+	+
	α-glucuronidase	−	−	−	−	−	−
	β-glucuronidase	−	−	−	−	−	−
	Glycosyltransferase	+	+	+	+	+	+
	Sugar transferase	+	+	+	+	+	+
Steroid core destructive enzyme	Bifunctional acetaldehyde-CoA/alcohol dehydrogenase	+	+	+	+	+	+
	3-hydroxyacyl-CoA dehydrogenase	+	+	+	+	+	+
Steroid side-chain oxidase	Acyl-CoA thioesterase	+	+	+	+	+	+
	Short chain dehydrogenase	+	+	+	+	+	+

## Data Availability

The authors declare that the data supporting the findings of this study are available within the paper. Should any raw data files be needed in another format, they are available from the corresponding author upon request.

## References

[1] Eyster K.M. (2016). The estrogen receptors: An overview from different perspectives. Methods Mol. Biol..

[2] Chen P., Li B., Ou-Yang L. (2022). Role of estrogen receptors in health and disease. Front. Endocrinol..

[3] Farage M., Maibach H. (2006). Lifetime changes in the vulva and vagina. Arch. Gynecol. Obstet..

[4] Muhleisen A.L., Herbst-Kralovetz M.M. (2016). Menopause and the vaginal microbiome. Maturitas.

[5] Kwon M.S., Lee H.K. (2022). Host and Microbiome Interplay Shapes the Vaginal Microenvironment. Front. Immunol..

[6] Cheskis B.J., Greger J.G., Nagpal S., Freedman L.P. (2007). Signaling by estrogens. J. Cell. Physiol..

[7] Biason-Lauber A., Lang-Muritano M. (2022). Estrogens: Two nuclear receptors, multiple possibilities. Mol. Cell. Endocrinol..

[8] Ingraham H.A., Herber C.B., Krause W.C. (2022). Running the female power grid across lifespan through brain estrogen signaling. Ann. Rev. Physiol..

[9] Hannan F.M., Elajnaf T., Vandenberg L.N., Kennedy S.H., Thakker R.V. (2023). Hormonal regulation of mammary gland development and lactation. Nat. Rev. Endocrinol..

[10] Knowlton A.A., Lee A.R. (2012). Estrogen and the cardiovascular system. Pharmacol. Ther..

[11] Baudry M., Bi X., Aguirre C. (2013). Progesterone-estrogen interactions in synaptic plasticity and neuroprotection. Neuroscience.

[12] Mauvais-Jarvis F., Clegg D.J., Hevener A.L. (2013). The role of estrogens in control of energy balance and glucose homeostasis. Endocr. Rev..

[13] Smy L., Straseski J.A. (2018). Measuring estrogens in women, men, and children: Recent advances 2012–2017. Clin. Biochem..

[14] Patel S., Homaei A., Raju A.B., Meher B.R. (2018). Estrogen: The necessary evil for human health, and ways to tame it. Biomed. Pharmacother..

[15] Yager J.D., Davidson N.E. (2006). Estrogen carcinogenesis in breast cancer. N. Engl. J. Med..

[16] Meyer M.R., Clegg D.J., Prossnitz E.R., Barton M. (2011). Obesity, insulin resistance and diabetes: Sex differences and role of oestrogen receptors. Acta Physiol..

[17] Walker S.E. (2011). Estrogen and autoimmune disease. Clin. Rev. Allergy Immunol..

[18] Barnabei V.M., Cochrane B.B., Aragaki A.K., Nygaard I., Williams R.S., McGovern P.G., Young R.L., Wells E.C., O’Sullivan M.J., Chen B. (2005). Menopausal symptoms and treatment-related effects of estrogen and progestin in the Women’s Health Initiative. Obstet. Gynecol..

[19] Au A., Feher A., McPhee L., Jessa A., Oh S., Einstein G. (2016). Estrogens, inflammation and cognition. Front. Neuroendocrinol..

[20] Coyoy A., Guerra-Araiza C., Camacho-Arroyo I. (2016). Metabolism Regulation by Estrogens and Their Receptors in the Central Nervous System Before and After Menopause. Horm. Metab. Res..

[21] Khan M.Z.I., Uzair M., Nazli A., Chen J.Z. (2022). An overview on estrogen receptors signaling and its ligands in breast cancer. Eur. J. Med. Chem..

[22] Carter A.E., Merriam S. (2023). Menopause. Med. Clin. N. Am..

[23] de Vos W.M., Tilg H., Van Hul M., Cani P.D. (2022). Gut microbiome and health: Mechanistic insights. Gut.

[24] Wang J., Li Z., Ma X., Du L., Jia Z., Cui X., Yu L., Yang J., Xiao L., Zhang B. (2021). Translocation of vaginal microbiota is involved in impairment and protection of uterine health. Nat. Comm..

[25] Minton K. (2023). Immune checkpoint blockade breaches the mucosal firewall to induce gut microbiota translocation. Nat. Rev. Immunol..

[26] Spor A., Koren O., Ley R. (2011). Unravelling the effects of the environment and host genotype on the gut microbiome. Nat. Rev. Microbiol..

[27] Pigrau M., Rodiño-Janeiro B.K., Casado-Bedmar M., Lobo B., Vicario M., Santos J., Alonso-Cotoner C. (2016). The joint power of sex and stress to modulate brain-gut-microbiota axis and intestinal barrier homeostasis: Implications for irritable bowel syndrome. Neurogastroenterol. Motil..

[28] Weersma R.K., Zhernakova A., Fu J. (2020). Interaction between drugs and the gut microbiome. Gut.

[29] Priya S., Burns M.B., Ward T., Mars R.A.T., Adamowicz B., Lock E.F., Kashyap P.C., Knights D., Blekhman R. (2022). Identification of shared and disease-specific host gene-microbiome associations across human diseases using multi-omic integration. Nat. Microbiol..

[30] Thriene K., Michels K.B. (2023). Human Gut Microbiota Plasticity throughout the Life Course. Int. J. Environ. Res. Public Health.

[31] Flores R., Shi J., Fuhrman B., Xu X., Veenstra T.D., Gail M.H., Gajer P., Ravel J., Goedert J.J. (2012). Fecal microbial determinants of fecal and systemic estrogens and estrogen metabolites: A cross-sectional study. J. Transl. Med..

[32] Baker J.M., Al-Nakkash L., Herbst-Kralovetz M.M. (2017). Estrogen–gut microbiome axis: Physiological and clinical implications. Maturitas.

[33] Clabaut M., Suet A., Racine P.J., Tahrioui A., Verdon J., Barreau M., Maillot O., Le Tirant A., Karsybayeva M., Kremser C. (2021). Effect of 17β-estradiol on a human vaginal *Lactobacillus crispatus* strain. Sci. Rep..

[34] Raftogianis R., Creveling C., Weinshilboum R., Weisz J. (2000). Estrogen metabolism by conjugation. JNCI Monogr..

[35] Elmassry M.M., Kim S., Busby B. (2021). Predicting drug-metagenome interactions: Variation in the microbial β-glucuronidase level in the human gut metagenomes. PLoS ONE.

[36] Neuman H., Debelius J.W., Knight R., Koren O. (2015). Microbial endocrinology: The interplay between the microbiota and the endocrine system. FEMS Microbiol. Rev..

[37] Hur H.J., Jeong Y.H., Lee S.H., Sung M.J. (2020). Quercitrin ameliorates hyperlipidemia and hepatic steatosis in ovariectomized mice. Life.

[38] Plottel C.S., Blaser M.J. (2011). Microbiome and malignancy. Cell Host Microb..

[39] Kitawaki J., Kado N., Ishihara H., Koshiba H., Kitaoka Y., Honjo H. (2002). Endometriosis: The pathophysiology as an estrogen-dependent disease. J. Steroid Biochem. Mol. Biol..

[40] Mori T., Kitawaki J. (2022). Role of estrogen and estrogen-related factors in endometriosis. Immunology of Endometriosis.

[41] Oliva M.M., Gambioli R., Forte G., Porcaro G., Aragona C., Unfer V. (2022). Unopposed estrogens: Current and future perspectives. Eur. Rev. Med. Pharmacol. Sci..

[42] Hu S., Ding Q., Zhang W., Kang M., Ma J., Zhao L. (2023). Gut microbial beta-glucuronidase: A vital regulator in female estrogen metabolism. Gut Microbes.

[43] Parida S., Sharma D. (2019). The Microbiome-Estrogen Connection and Breast Cancer Risk. Cells.

[44] Ervin S.M., Li H., Lim L., Roberts L.R., Liang X., Mani S., Redinbo M.R. (2019). Gut microbial β-glucuronidases reactivate estrogens as components of the estrobolome that reactivate estrogens. J. Biol. Chem..

[45] Salliss M.E., Farland L.V., Mahnert N.D., Herbst-Kralovetz M.M. (2021). The role of gut and genital microbiota and the estrobolome in endometriosis, infertility and chronic pelvic pain. Hum. Reprod. Updat..

[46] Qin R., Tian G., Liu J., Cao L. (2022). The gut microbiota and endometriosis: From pathogenesis to diagnosis and treatment. Front. Cell Infect. Microbiol..

[47] Soto A., Martín V., Jiménez E., Mader I., Rodríguez J.M., Fernández L. (2014). Lactobacilli and bifidobacteria in human breast milk: Influence of antibiotherapy and other host and clinical factors. J. Pediatr. Gastroenterol. Nutr..

[48] Martín V., Cárdenas N., Ocaña S., Marín M., Arroyo R., Beltrán D., Badiola C., Fernández L., Rodríguez J.M. (2019). Rectal and Vaginal Eradication of *Streptococcus agalactiae* (GBS) in Pregnant Women by Using *Lactobacillus salivarius* CECT 9145, A Target-specific Probiotic Strain. Nutrients.

[49] Xu X., Roman J.M., Issaq H.J., Keefer L.K., Veenstra T.D., Ziegler R.G. (2007). Quantitative measurement of endogenous estrogens and estrogen metabolites in human serum by liquid Chromatography–Tandem mass spectrometry. Anal. Chem..

[50] Zhao X., Wang Y., Xu X., Tian K., Zhou D., Meng F., Huo H. (2020). Genomics analysis of the steroid estrogen-degrading bacterium *Serratia nematodiphila* DH-S01. Biotechnol. Biotechnol. Equip..

[51] Qiu Q., Wang P., Kang H., Wang Y., Tian K., Huo H. (2019). Genomic analysis of a new estrogen-degrading bacterial strain, *Acinetobacter* sp. DSSKY-A-001. Int. J. Gen..

[52] Baele M., Baele P., Vaneechoutte M., Storms V., Butaye P., Devriese L.A., Verschraegen G., Gillis M., Haesebrouck F. (2000). Application of tRNA intergenic spacer PCR for identification of *Enterococcus* species. J. Clin. Microbiol..

[53] Sui Y., Wu J., Chen J. (2021). The role of gut microbial β-glucuronidase in estrogen reactivation and breast cancer. Front. Cell Dev. Biol..

[54] Szentirmai A. (1990). Microbial physiology of sidechain degradation of sterols. J. Ind. Microbiol..

[55] Ge H., Yang L., Li B., Feng Y., Wang S., Zheng Y., Feng L., Liu Y., Du Z., Zhang L. (2021). A comparative study on the biodegradation of 17β-estradiol by *Candida utilis* CU-2 and *Lactobacillus casei* LC-1. Front. Energy Res..

[56] Bragin E.Y., Shtratnikova V.Y., Dovbnya D.V., Schelkunov M.I., Pekov Y.A., Malakho S.G., Egorova O.V., Ivashina T.V., Sokolov S.L., Ashapkin V.V. (2013). Comparative analysis of genes encoding key steroid core oxidation enzymes in fast-growing *Mycobacterium* spp. strains. J. Steroid Biochem. Mol. Biol..

[57] Shi J., Fujisawa S., Nakai S., Hosomi M. (2004). Biodegradation of natural and synthetic estrogens by nitrifying activated sludge and ammonia-oxidizing bacterium *Nitrosomonas europaea*. Water Res..

[58] Donova M.V., Egorova O.V. (2012). Microbial steroid transformations: Current state and prospects. Appl. Microbiol. Biotechnol..

[59] Xiong W., Yin C., Wang Y., Lin S., Deng Z., Liang R. (2020). Characterization of an efficient estrogen-degrading bacterium *Stenotrophomonas maltophilia* SJTH1 in saline-, alkaline-, heavy metal-contained environments or solid soil and identification of four 17β-estradiol-oxidizing dehydrogenases. J. Hazard. Mat..

[60] Wang P., Zheng D., Liang R. (2019). Isolation and characterization of an estrogen-degrading *Pseudomonas putida* strain SJTE-1. 3 Biotech.

[61] Yu C.P., Roh H., Chu K.H. (2007). 17β-Estradiol-degrading bacteria isolated from activated sludge. Environ. Sci. Technol..

[62] Chen Y.L., Yu C.P., Lee T.H., Goh K.S., Chu K.H., Wang P.H., Ismail W., Shih C.J., Chiang Y.R. (2017). Biochemical mechanisms and catabolic enzymes involved in bacterial estrogen degradation pathways. Cell Chem. Biol..

[63] Bilal M., Barceló D., Iqbal H.M. (2021). Occurrence, environmental fate, ecological issues, and redefining of endocrine disruptive estrogens in water resources. Sci. Total Environ..

[64] Hernandez-Raquet S.C.G. (2010). Occurrence, fate and biodegradation of estrogens in sewage and manure. Appl. Microbiol. Biotechnol..

[65] Wieacker P. (2009). Genetic Aspects of premature ovarian failure. J. Reprod. Med. Endocrinol..

[66] Liang J., Shang Y. (2013). Estrogen and cancer. Ann. Rev. Physiol..

[67] Bulun S.E., Yang S., Fang Z., Gurates B., Tamura M., Sebastian S. (2002). Estrogen production and metabolism in endometriosis. Ann. N. Y. Acad. Sci..

[68] Van Der Veer C., Hertzberger R.Y., Bruisten S.M., Tytgat H.L., Swanenburg J., Angelino-Bart A., Schuren F., Molenaar D., Reid G., de Vries H. (2019). Comparative genomics of human *Lactobacillus crispatus* isolates reveals genes for glycosylation and glycogen degradation: Implications for in vivo dominance of the vaginal microbiota. Microbiome.

[69] Lv B., Sun H., Huang S., Feng X., Jiang T., Li C. (2018). Structure-guided engineering of the substrate specificity of a fungal β-glucuronidase toward triterpenoid saponins. J. Biol. Chem..

[70] Coulon S., Chemardin P., Gueguen Y., Arnaud A., Galzy P. (1998). Purification and characterization of an intracellular β-glucosidase from *Lactobacillus casei* ATCC 393. Appl. Biochem. Biotechnol..

[71] Dabek M., McCrae S.I., Stevens V.J., Duncan S.H., Louis P. (2008). Distribution of β-glucosidase and β-glucuronidase activity and of β-glucuronidase gene gus in human colonic bacteria. FEMS Microbiol. Ecol..

[72] Muccee F., Ghazanfar S., Ajmal W., Al-Zahrani M. (2022). In-Silico Characterization of Estrogen Reactivating β-Glucuronidase Enzyme in GIT Associated Microbiota of Normal Human and Breast Cancer Patients. Genes.

[73] Mroczynska M., Libudzisz Z. (2010). Beta-glucuronidase and beta-glucosidase activity of Lactobacillus and Enterococcus isolated from human feces. Pol. J. Microbiol..

[74] Landete J.M., Arqués J., Medina M., Gaya P., de Las Rivas B., Muñoz R. (2016). Bioactivation of phytoestrogens: Intestinal bacteria and health. Crit. Rev. Food Sci. Nut..

[75] Beck A.P., Li H., Ervin S.M., Redinbo M.R., Mani S. (2019). Inhibition of Microbial Beta-Glucuronidase Does Not Prevent Breast Carcinogenesis in the Polyoma Middle T Mouse. bioRxiv.

[76] Nakamura J., Kubota Y., Miyaoka M., Saitoh T., Mizuno F., Benno Y. (2002). Comparison of four microbial enzymes in Clostridia and Bacteroides isolated from human feces. Microbiol. Immunol..

